# Reproductive Strategy Inferred from Major Histocompatibility Complex-Based Inter-Individual, Sperm-Egg, and Mother-Fetus Recognitions in Giant Pandas (*Ailuropoda melanoleuca*)

**DOI:** 10.3390/cells8030257

**Published:** 2019-03-19

**Authors:** Ying Zhu, Qiu-Hong Wan, He-Min Zhang, Sheng-Guo Fang

**Affiliations:** 1 MOE Key Laboratory of Biosystems Homeostasis & Protection, State Conservation Centre for Gene Resources of Endangered Wildlife, College of Life Sciences, Zhejiang University, Hangzhou 310058, China; so_zy2003@126.com (Y.Z.); qiuhongwan@zju.edu.cn (Q.-H.W.); 2 China Conservation and Research Center for the Giant Panda, No. 98 Tongjiang Road, Dujiangyan 611800, China; panda_zhanghm@163.com

**Keywords:** MHC-I- and MHC-II-dependent inter-individual recognition, MHC-II-associated sperm-egg recognition, MHC-I-based mother-fetus recognition, giant panda, long-fragment super haplotype

## Abstract

Few major histocompatibility complex (MHC)-based mate choice studies include all MHC genes at the inter-individual, sperm-egg, and mother-fetus recognition levels. We tested three hypotheses of female mate choice in a 17-year study of the giant panda (*Ailuropoda melanoleuca*) while using ten functional MHC loci (four MHC class I loci: *Aime*-C, *Aime*-F, *Aime*-I, and *Aime*-L; six MHC class II loci: *Aime*-DRA, *Aime*-DRB3, *Aime*-DQA1, *Aime*-DQA2, *Aime*-DQB1, and *Aime*-DQB2); five super haplotypes (SuHa, SuHaI, SuHaII, DQ, and DR); and, seven microsatellites. We found female choice for heterozygosity at *Aime*-C, *Aime*-I, and DQ and for disassortative mate choice at *Aime*-C, DQ, and DR at the inter-individual recognition level. High mating success occurred in MHC-dissimilar mating pairs. No significant results were found based on any microsatellite parameters, suggesting that MHCs were the mate choice target and there were no signs of inbreeding avoidance. Our results indicate *Aime*-DQA1- and *Aime*-DQA2-associated disassortative selection at the sperm-egg recognition level and a possible *Aime*-C- and *Aime*-I-associated assortative maternal immune tolerance mechanism. The MHC genes were of differential importance at the different recognition levels, so all of the functional MHC genes should be included when studying MHC-dependent reproductive mechanisms.

## 1. Introduction

Understanding the genetic basis and the driving forces of mate choice in animals has always been a major goal of evolutionary ecologists [1,2]. It has been proposed that females prefer males who can maximize their reproductive success and increase offspring quality/fitness [3,4]. Immunocompetence is undoubtedly an essential index of an individual’s fitness [5,6] and major histocompatibility complex (MHC) genes are suitable candidates in investigating the genetic basis underlying mate choice decisions, as MHC molecules can recognize and present antigens to T cells and trigger immune reactions [7,8,9,10]. A growing body of evidence shows the association between pathogen resistance and MHC haplotypes or alleles [11,12,13,14]. 

In recent years, an increasing number of studies have focused on MHC-associated mate choice, including three non-exclusive hypotheses that could explain the MHC-based mate choice. Firstly, heterozygous advantage: according to this hypothesis, the choosy sex could obtain additive benefits from mating with MHC-heterozygous mates whose disease resistance might be inherited by their offspring [15,16]. For example, in tuco-tucos (*Ctenomys* spp.), females prefer MHC-heterozygous males [17]. In the scarlet rosefinch (*Carpodacus erythrinus*), social males with low MHC heterozygosity are cheated on by their females more frequently than highly MHC-heterozygous males [18]. Secondly, the genetic compatibility hypothesis: the choosy sex is assumed to select MHC-dissimilar partners, resulting in the production of offspring with diverse genotypes that can recognize a broad array of pathogens and hence increase their fitness [15,16,19]. In the grey mouse lemur (*Microcebus murinus*), the fathers have lower allele sharing and a greater amino acid distance to the mother than the randomly assigned males [20]. A study on blue petrels (*Halobaena caerulea*) revealed that females mated more with functionally (not evolutionary) MHC-dissimilar males than with random males [21]. Similar patterns have been observed in great frigatebirds (*Fregata minor*) [22], the Chinese rose bitterling (*Rhodeus ocellatus*) [23], the pot-bellied seahorse (*Hippocampus abdominalis*) [24], the fat-tailed dwarf lemur (*Cheirogaleus medius*) [25], and mandrills (*Mandrillus* spp.) [26]. Furthermore, to acquire the best immunogenetic composition for their offspring, females would choose mates with the most appropriate MHC diversity (maximum or intermediate), which is also referred to as the optimal hypothesis, and it is an extension of the genetic compatibility hypothesis [10]. Studies in sticklebacks (family Gasterosteidae) have found that females with many alleles prefer males with few alleles, and vice versa, in order to obtain an optimal level of MHC diversity in their offspring for resistance against parasites and pathogens [27,28]. Thirdly, the inbreeding avoidance hypothesis: the choosy sex is expected to seek dissimilar partners, not only with regard to MHC, but also genome-wide, in order to gain fitness benefits [29,30].

Mate choice results from non-random reproductive investment, which could happen at the precopulatory stage or/and at the postcopulatory stage. In the precopulatory stage (or individual recognition level), the choosy sex uses visual, acoustic, or odor cues to choose mates [15]. In the postcopulatory stage, the females increase offspring quality/fitness by their eggs differentiating between sperm during fertilization (sperm-egg recognition), and by following a differential allocation strategy during embryo implantation (mother-fetus recognition) [16]. Human leukocyte antigen (HLA) class I and class II molecules are expressed on sperm cell surfaces [31,32,33], making the complementary cryptic female choice for MHC genotypes possible at the gamete level. Sperm selection that targets different levels of MHC diversity has been reported in several species. In some rodent species, females refuse to accept MHC-similar sperm [34], and in humans, females that mate with a male who has the same HLA haplotype tend to have a greater chance of spontaneous abortion [35,36,37]. In red junglefowl (*Gallus gallus*), the eggs favor sperm that is from MHC-dissimilar males [38]. In contrast, a fertilization advantage for MHC-similar mates has been observed in Atlantic salmon (*Salmo salar*) [39], Chinook salmon (*Oncorhynchus tshawytscha*) [40], and guppies (*Poecilia reticulata*) [41]. A recent study of the three-spined stickleback (*Gasterosteus aculeatus*) revealed that sperm selection resulted in offspring with an intermediate level of MHC diversity [42]. 

The giant panda (*Ailuropoda melanoleuca*) is an endangered species and its captive breeding has always been focused in China. Giant pandas usually have polyandrous/polygynous multiple mating systems [43]. Females are very choosy, and they have significantly higher copulation and birth rates when paired with preferred males [44]. In breeding programs, natural mating as well as artificial insemination are adopted to increase reproductive success [45]. Although the number of female giant pandas that are available to breed has increased, the fertilization rate is still low (~50% for nearly 20 years). Furthermore, female giant pandas have spontaneous abortions when they are inseminated by sperm from a male that they do not like [46]. Therefore, there may be an MHC-based mechanism that determines mate choice and fertilization in giant pandas. 

It is best to investigate all MHC genes concerning mate choice, as MHC class I and class II molecules mainly present intracellular and extracellular pathogen-derived antigens, respectively [9], and the selection of MHC genes might influence mate choice results [47]. Few studies have focused on a large region of the MHC due to a lack of structural knowledge and an effective genotyping method [48], including a giant panda mate choice study that only used three MHC class II genes [49]. In previous studies, we characterized six functional MHC class II genes (*Aime*-DRA, *Aime*-DRB3, *Aime*-DQA1, *Aime*-DQA2, *Aime*-DQB1, and *Aime*-DQB2) [50,51] and four classical MHC class I genes (*Aime*-C, *Aime*-F, *Aime*-I, and *Aime*-L) [52], and we developed their genotyping protocols in the giant panda. Therefore, our previous studies provide a good foundation to investigate the relationship between a large number of MHC genes and female choice at the inter-individual, sperm-egg, and mother-fetus recognition levels in giant pandas. 

In the present study, we took advantage of multiple years of observations and the possession of genetic data of a captive population in the Wolong Chinese Research and Conservation Center for the Giant Panda. We aimed to: (1) test three mate choice hypotheses at the individual recognition level (MHC-heterozygous choice, MHC-compatibility choice, and inbreeding avoidance) while using 10 MHC genes and seven microsatellites. If the heterozygote advantage is the main driving force of female mate choice, more diverse males should be preferred than less diverse ones, regardless of the females’ MHC genotypes. If female choice favors the production of offspring with high fitness that is based on MHC compatibility, we expect that females would choose MHC-dissimilar partners that provide the most appropriate level of MHC dissimilarity (maximum or intermediate) in the offspring. If mate choice aims to avoid inbreeding, then we expect genome-wide dissimilarity between partners (MHC and microsatellites); (2) test whether cryptic female choice for MHC compatibility occurs at the gamete level; and, (3) characterize successful embryo implantations by comparing the MHC genotypes of mothers and their offspring.

## 2. Materials and Methods

### 2.1. Study Species and Behavioral Observations of Inter-Individual Recognition

Giant pandas were housed in the Wolong Chinese Research and Conservation Center for the Giant Panda. Female giant pandas come into estrus from February to June [43], when their appetite decreases, males’ urination frequency increases, and they rub their genitalia against the ground or walls [43,46]. Two rutting females and males are usually put into two adjacent cages. Neighboring pandas have access to each other through a cage fence with full sight, hearing, and smell, but limited touch. Female pandas face multiple males that are consecutively presented. Veterinarians use pre-mating behaviors to determine when the males are sent to females for mating. If a female is interested in a male, then they usually respond by sniffing and pushing the fence, before the male is sent to the female’s cage to copulate. If a female is uninterested, then she does not respond or behaves aggressively. Female mate choice at the inter-individual recognition level was determined by naturally analyzing mating pairs that were recorded in a studbook [53]. There were 33 females and 21 males in the breeding program from 1991 and 2008, except for 1994, but we failed to obtain samples from five females and four males. Therefore, for the inter-individual recognition study, we included 182 natural mating events that involved 28 females and 17 males.

### 2.2. Sperm-Egg and Mother-Fetus Recognition

Female giant pandas have an annual estrus cycle with spontaneous ovulation [54,55]. They naturally mate with multiple males and require artificial insemination with males that they mate with and with males that they do not mate with but have good-quality sperm. Sperm from all males have a chance to access the egg, but only one of them is successful through a mechanism at the gamete stage, and sperm-egg recognition may occur at the gamete stage. Over 17 years, a total of 80 offspring (zygotes observed) were produced. Other zygotes were combinations of egg-to-sperm haplotypes, except for offspring haplotypes. The information that is required for natural mating and artificial insemination was acquired from the studbook and SPARKS 1.5 [53]. 

In addition, we compared the difference between the zygotes observed and other zygotes (combinations of egg-to-sperm haplotypes, except for offspring haplotypes) to mothers in successful embryo implantation events, and called it the “mother-fetus recognition level”.

### 2.3. DNA Extraction, MHC and Microsatellite Genotyping, and the Definition of a Super Haplotype

We collected 110 blood and the fecal samples. Blood samples were obtained during a routine medical examination and preserved in liquid nitrogen. Fecal samples were less than two days old and stored in 95% ethanol. Genomic DNA extraction from the blood and fecal samples was conducted, as described by Wan et al. [56].

We used seven microsatellite loci (*Aim*-3, *Aim*-5, *Aim*-10, *Aim*-11, *Aim*-13, *Aim*-14, and *Aim*-16) that performed well in a previous paternity test [57] to conduct paternity analysis for 113 individuals. Polymerase chain reaction (PCR) amplification and genotyping mirrored that described by Zhang et al. [58].

We used 10 functional *Aime*-MHC loci, including four class I loci (*Aime*-C, *Aime*-F, *Aime*-I, and *Aime*-L) and six class II loci (*Aime*-DRA, *Aime*-DRB3, *Aime*-DQA1, *Aime*-DQA2, *Aime*-DQB1, and *Aime*-DQB2). We genotyped all individuals at the polymorphic MHC class I exon 2–3 regions and MHC class II exon 2. The primer sets, PCR amplification, and genotyping were as described in two previous studies [52,59].

A physical MHC map revealed that as well as four MHC class I genes, there were also six MHC class II genes linked together [52,60]. In addition to using the above-mentioned independent MHC loci, we analyzed the allele linkage relationships of four MHC class I genes, six MHC class II genes, all MHC genes, genes in the DQ region, and genes in the DR region. For example, we identified homozygotes in four MHC class I genes of the offspring and then inferred the linkage relationship between the mother and father according to Mendel’s law. We named the linkage of four MHC class I genes as SuperHaplotypeI (SuHaI). Using the same procedure, we named the linkage relationships between six MHC class II genes and all MHC genes SuHaII and SuHa, respectively. Genes in the DQ and DR regions were simply called DQ and DR, respectively.

### 2.4. Paternity Test

We performed a paternity analysis with seven microsatellite loci while using an exclusive method that was based on Mendel’s law, and MHC genotype data confirmed the results. Information on mothers was obtained from the studbook [53].

### 2.5. Data Analysis

#### 2.5.1. Female Mate Choice at the Inter-Individual Recognition Level

Three mate choice hypotheses, heterozygote advantage, genetic compatibility, and inbreeding avoidance were tested at the inter-individual recognition level.

We tested whether females prefer heterozygous males by utilizing three parameters: (1) The number of heterozygotes (*H*_obs_) at MHC loci in males. We used 0 and 1 to represent homozygote and heterozygote, respectively; (2) Multilocus heterozygosity (MLH) in males, i.e., the proportion of heterozygous microsatellite loci that accounted for the total number of microsatellite loci; (3) *d*^2^ value (microsatellites), i.e., the genetic distance between the two alleles. We calculated the *d*^2^ for each individual according to the formula *d*^2^ = 1/n Σ^n^ (a_i_ − a_j_)^2^, where a_i_ and a_j_ refer to the repeat length of two individual alleles [61].

We tested whether female giant pandas prefer MHC-dissimilar or -similar males (choice for genetic compatibility) with three parameters: (1) The number of MHC alleles that is shared between females and males as “Nas”, which is twice the number of alleles that is shared by females and males divided by the total number of females and males [62]; (2) The pairwise functional amino acid distance between MHC alleles of females and males, calculated as “Faadis” = D_ab_ + D_aB_ + D_AB_ + D_ab_, where A, a, B, and b are four alleles in two mates [63]. Each amino acid was characterized by five physicochemical variables: z1 (hydrophobicity), z2 (steric bulk), z3 (polarity), and z4 and z5 (electronic effects) [64]. The functional amino acid distance was the Euclidean metric of the two vectors, which consists of two alleles from a female and male [65]. We not only considered the distance at all sites, but also at the antigen binding sites (ABSs), as it is ABSs that determine pathogen binding and recognition and they are considered a functional region [14,66,67]. We defined ABSs according to human sequences [68]. 

We tested the inbreeding avoidance hypothesis using Queller and Goodnight’s relatedness (microsatellites) [69], as well as Nas and Faadis. Queller and Goodnight’s relatedness was calculated in SPAGeDi v 1.4 [70] to estimate the genetic similarity between female and male giant pandas. If mate choice aims to avoid inbreeding, we would expect to see choice that is based on compatibility at the genome level (MHC and microsatellites), or female mate choice was targeted to MHCs. 

We adopted three approaches to test each hypothesis. Firstly, we performed a randomization test, which is a nonparametric approach that is based on Monte Carlo sampling, to test whether female giant pandas randomly choose their partners for mating. We simulated the natural mating scenario (same accessible males for each female in the respective year), with the exception that females randomly chose males. We generated a null distribution by allowing each female to randomly choose 10,000 times between all males in the respective year. We then compared the mean of the values that were obtained from 182 naturally mated pairs or males involved in natural mating with a set of randomly matched pairs. We calculated the exact *p* values as twice the proportion of the simulations, which gave higher values than those observed. If females preferred heterozygous males, we expected that the mean values (*H*_obs_, MLH, and *d*^2^) of males that were naturally mated would be significantly higher than those of the randomly assigned males. If females preferred MHC-dissimilar males, we expected that the mean Nas of naturally mated pairs would be significantly lower than that of the randomly assigned pairs, or that the Faadis of naturally mated pairs would be significantly higher than that of the randomly assigned pairs. If females aim to avoid inbreeding, then we expected that the mean values (Faadis and relatedness) would be higher than those of randomly assigned pairs. The randomization tests were conducted in ResamplingStats v 4.0 (ResamplingStats Inc., Arlington, TX, USA).

Secondly, we used a paired Student’s *t*-test (or Wilcoxon test if the model assumptions were not met) to test whether the natural mating group exhibited higher heterozygosity and/or greater MHC divergence than non-mating group by comparing the mean values of males that are involved in natural mating and those of males that were not involved in mating. *H*_obs_, MLH, and *d*^2^ were used to test the heterozygote advantage hypothesis, and Nas, Faadis, and relatedness were used to test the compatibility and inbreeding avoidance hypotheses. 

We employed a model-based approach to explore the association between genetic variables and the probability of natural mating success. We performed a generalized linear mixed model (GLMM) with a binomial distribution and logic link function for 956 paring events, and the dependent variable was coded as 1 for a natural mating event and 0 for those that did not. The MHC genetic variables (*H*_obs_, Nas, and Faadis) were included as the fixed effects and female ID (to avoid female pseudoreplication), male ID, and year (to account for differences among breeding years) were included as random effects. 

#### 2.5.2. Sperm-Egg Recognition Level

We used a randomization test, a paired Student’s *t*-test, and a GLMM to test the MHC compatibility hypothesis at the sperm-egg recognition level.

Randomization was based on the data obtained (same males for fertilization or natural mating in the respective year), with the exception that the eggs randomly chose sperm. We generated a null distribution by allowing each egg to randomly choose 10,000 times between all of the sperm of the respective year. Subsequently, we compared the mean MHC divergence from the zygotes that were observed (Faadis = D_AB,_ where A and B are two haplotypes in two gametes, N = 65) with a distribution of egg-to-sperm divergence from randomly assigned zygotes. The exact *p* values were calculated, as described above. 

Paired Student *t*-tests (or Wilcoxon tests if the model assumptions were not met) were used to test whether the observed combination of eggs and sperm indicated greater MHC divergence by comparing the mean values of the zygotes observed (offspring) with other zygotes. 

We used a GLMM with a binomial distribution and logic link to test whether MHC divergence between eggs and sperm influenced breeding success (successful births). The dependent variable was coded as 1 for a breeding event and 0 for a non-breeding event. Egg-to-sperm MHC divergence (Faadis) was included as a fixed effect and female ID, male ID, and year were included as the random effects. 

The paired Student *t*-tests, Wilcoxon tests, and GLMMs were conducted in SPSS v. 20.0 (SPSS Inc., Chicago, IL, USA).

#### 2.5.3. Mother-Fetus Recognition Level

We used paired Student *t*-tests or Wilcoxon tests, as appropriate, to test whether the zygotes observed (offspring) had lower MHC divergence (Faadis) from the mother than other zygotes (combinations of egg-to-sperm haplotypes, except for offspring haplotypes) by comparing the mean Faadis of zygotes observed to the mother with other zygotes to the mother.

A GLMM with a binomial distribution and logic link was used to explore the association between genetic variables and the probability of breeding success. The dependent variable was coded as 1 for a breeding event and 0 for a non-breeding event. The MHC divergence of zygotes from the mother was included as a fixed effect, and the random effects mirrored those described above. 

We adjusted the *p* values with a false discovery rate of 20%, following the Benjamini–Hochberg procedure for multiple testing [71,72].

### 2.6. Declarations Ethics Statement 

All blood and fecal samples were collected from captive pandas that were housed in the Wolong Chinese Research and Conservation Center for the Giant Panda. Blood samples were collected during routine examinations with permission from the China Giant Panda Protection and Management Office. We obtained specific permission from the China Research and Conservation Center for the Giant Panda to take fecal samples from captive individuals during the non-breeding season. Permission to use the samples was given by the State Conservation Center for Gene Resources of Endangered Wildlife of China, where they were deposited.

## 3. Results

### 3.1. Microsatellite and MHC Diversity

The male MLH ranged from 0.286 to 1 (median = 0.714), *d*^2^ ranged from 0.714 to 41.571 (median = 25.857), and Queller and Goodnight’s relatedness ranged from −0.484 to 0.829 (median = −0.070).

A total of 47 MHC sequences were isolated, which are the same as the published sequences, and included 22 MHC class I alleles and 25 MHC class II alleles: seven *Aime*-C (*Aime*-C*01–08, JX987000–JX987005, and JX987007), one *Aime*-F (*Aime*-F*01 and JX9870008), seven *Aime*-I (*Aime*-I*01–07 and JX987009–JX987015), seven *Aime*-L (*Aime*-L*01–06 and JX987016–JX987021), seven DQA1 (DQA1*01–07), three DQA2 (DQA2*01–03), 6 DQB1 (DQB1*01–06), seven DRB3 (DRB3*01–05, 07–08), one DRA, and one DQB2. We obtained only one allele at *Aime*-F, *Aime*-DRA, and *Aime*-DQB2, so we excluded these three loci from the analysis.

Furthermore, we obtained 36 SuHa, 20 SuHaI, 18 SuHaII, 18 DQ, and seven DR. The linkage relationships of SuHaII, SuHaI, SuHa, and DQ are presented in Figures S1–S4, respectively. The number of super haplotypes in the DR region was the same as the allele number at DRB3, as there were only two loci in the DR region, with DRA being a homozygote. 

The mean number of variable amino acid sites in MHC class II genes was higher than that in MHC class I genes with respect to the whole exon and all of the ABS sites (12.8% vs. 11.5%, respectively, and 41.0% vs. 25.7%, respectively). This was found by comparing the variable amino acid sites in SuHaI and SuHaII (Table S1). However, the mean difference between pairwise alleles in MHC class I genes was greater than that in MHC class II genes (Table S1), as was the case for SuHaI and SuHaII. Moreover, the number of variable amino acid sites and the difference between the pairwise alleles in the DR region were both greater than those in the DQ region. 

### 3.2. Inter-Individual Recognition

The number of males that were accessible to females ranged from three to 11 during the 17-year period, and females naturally mated with 1–4 males.

#### 3.2.1. Heterozygosity Advantage

##### Males Involved in Natural Mating Versus Randomly Assigned Males

**Figure 1:**
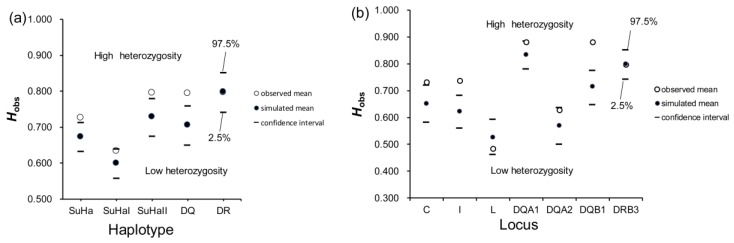
Mean individual major histocompatibility complex heterozygosity (*H*_obs_) of males involved in natural mating and randomly assigned males. (**a**) five super haplotypes; and, (**b**) seven individual loci. Two-tailed 95% confidence intervals are indicated by black lines.

Concerning super haplotypes, males that were involved in natural mating had significantly more heterozygotes at SuHa, SuHaII, and DQ than the randomly assigned males (*P*_SuHa_ = 0.009, *P*_SuHaII_ = 0.011, and *P*_DQ_ = 0.001; Figure 1a). Regarding individual loci, males that are involved in natural mating had more heterozygotes at *Aime*-C, *Aime*-I, and *Aime*-DQB1 than randomly assigned males (*P*_C_ = 0.020, *P*_I_ = 0.000, and *P*_DQB1_ = 0.000; Figure 1b). However, there were no significant differences in heterozygosity at any other super haplotype or locus between the males that naturally mated and randomly assigned males (Figure 1). Furthermore, we found no significant difference in MLH or *d*^2^ between males that naturally mated and those that were randomly assigned (Table 1).

##### Males Involved in Natural Mating Versus Natural Non-Mating Males

**Figure 2:**
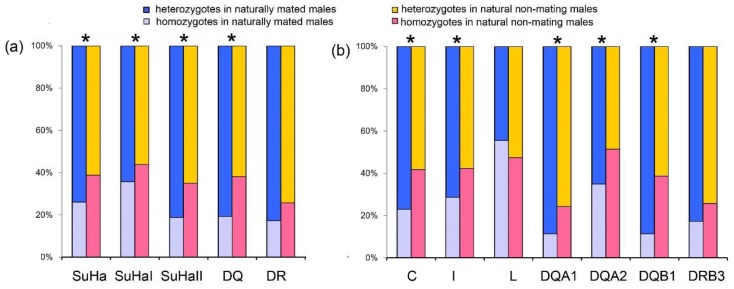
Proportions of heterozygotes and homozygotes in males involved in natural mating and natural non-mating males. (**a**) five super haplotypes; and, (**b**) seven individual loci. Asterisk shows results with *p* values that are smaller than 0.05.

Males that were involved in natural mating had a higher proportion of heterozygotes at SuHa, SuHaI, SuHaII, and SuHaDQ than those natural non-mating males (males not involved in mating, *P*_SuHa_ = *P*_SuHaI_ = *P*_SuHaII_ = *P*_DQ_ = 0.000; Figure 2a). This pattern was found at all loci, except *Aime*-L and *Aime*-DRB (*P*_C_ = 0.000, *P*_I_ = 0.000, *P*_DQA1_ = 0.001, *P*_DQA2_ = 0.007, and *P*_DQB1_ = 0.000; Figure 2b). We found no significant differences in MLH or *d*^2^ between males that were involved in natural mating and other males (Table 1).

##### Relationship between Male MHC Heterozygosity and Natural Mating Success

Being encouraged by the results above, we combined seven polymorphic MHC loci (DQA1, DQA2, DQB1, DRB3, *Aime*-C, *Aime*-I, and *Aime*-L) to model the relationship between male MHC heterozygosity and natural mating success, and found a nonsignificant relationship between overall male MHC heterozygosity and natural mating success (F = 2.963, *df* = 954, *P* = 0.86). Female ID, male ID, and year did not have any effect on natural mating success. 

#### 3.2.2. Genetic Compatibility and Inbreeding Avoidance

##### Naturally Mated Pairs versus Randomly Assigned Pairs

**Figure 3:**
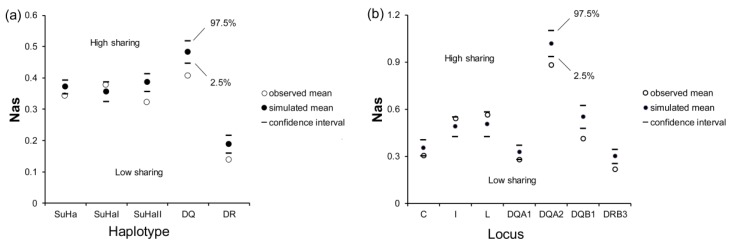
Mean allele sharing values (Nas) in females and males involved in natural mating and in females and randomly assigned males. (**a**) five super haplotypes; and, (**b**) seven individual loci. Two-tailed 95% confidence intervals are indicated by black lines.

There was a significant difference in Nas at SuHa, SuHaII, DQ, and DR between naturally mated pairs and randomly assigned pairs (*P*_SuHa_ = 0.006, *P*_SuHaII_ = 0.000, *P*_DQ_ = 0.000, and *P*_DR_ = 0.000; Figure 3a). Males that were involved in natural mating had significantly lower Nas at *Aime*-DQA1, DQA2, DQB1, and DRB3 than the randomly assigned pairs (*P*_DQA1_ = 0.038, *P*_DQA2_ = 0.001, *P*_DQB1_ = 0.000, and *P*_DRB3_ = 0.000; Figure 3b). However, we did not find any significant differences in Nas at SuHaI or other loci (Figure 3). 

Naturally mated pairs had significantly higher Faadis at SuHa, SuHaII, DQ, DR, *Aime*-C, DQA1, DQA2, DQB1, and DRB3 than randomly assigned pairs with respect to all sites and the ABS sites (Table 2). However, we did not find a significant difference in Faadis at SuHaI, *Aime*-I, or *Aime*-L between the pairs with respect to all sites and ABS sites (Table 2). In addition, there was no significant difference between the naturally mated pairs and randomly assigned pairs in genetic relatedness at microsatellites (Table 1).

##### Naturally Mated Pairs versus Non-Mating Pairs 

**Figure 4:**
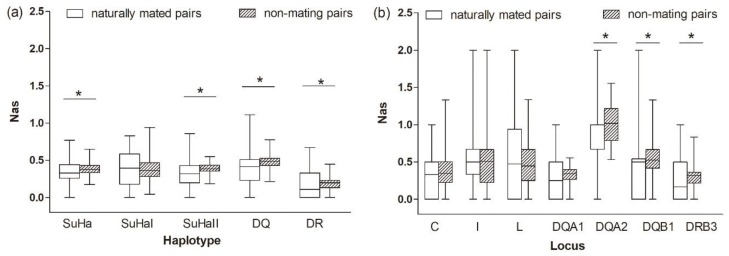
Proportion of allele sharing (Nas) in naturally mated pairs and non-mating pairs (**a**) five super haplotypes, and (**b**) seven individual loci. Asterisk shows results with *p* values that are smaller than 0.05.

**Figure 5:**
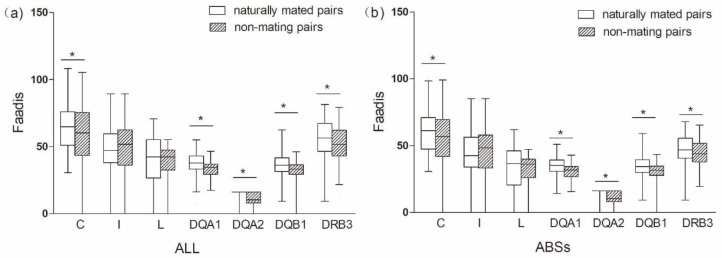
Functional amino acid distances (Faadis) in naturally mated pairs and non-mating pairs. (**a**) functional amino acid distance of all sites; and, (**b**) functional amino acid distance of antigen binding sites (ABSs). Asterisk shows results with *p* values that are smaller than 0.05.

The comparison between naturally mated pairs and non-mating pairs that are based on allele sharing, functional amino acid distances, and genetic relatedness was the same as between naturally mated pairs and randomly assigned pairs (Figure 4 and Figure 5 and Table 1 and Table 3). We found no evidence to support the inbreeding avoidance hypothesis when comparing the results of the MHC genes (significant) and microsatelites (nonsignificant).

##### Relationship between MHC Divergence between Females and Males and Natural Mating Success

**Figure 6:**
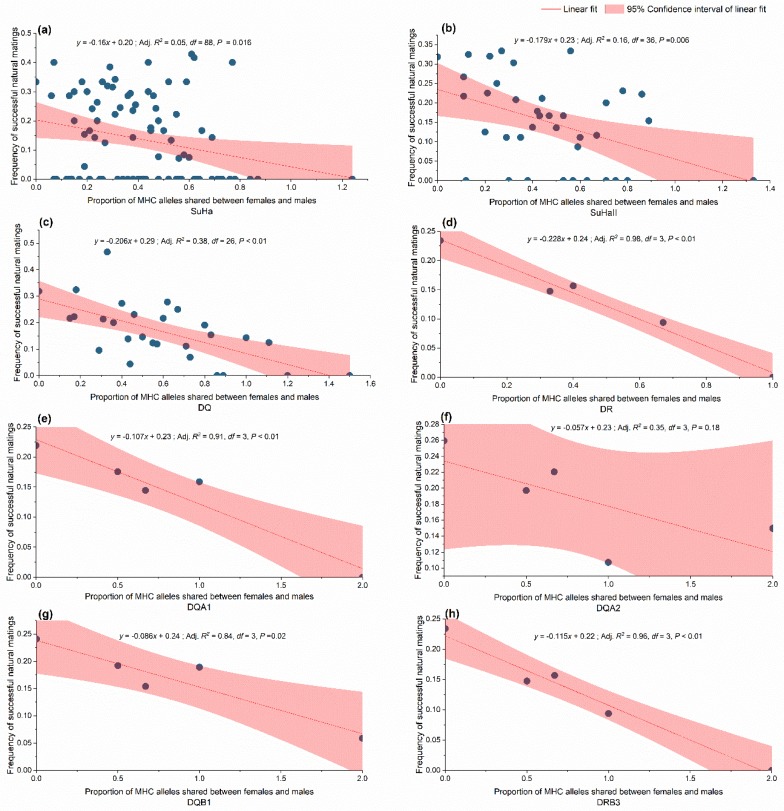
Relationship between shared major histocompatibility complex (MHC) alleles and frequency of successful natural mating. (**a**) SuHa, including four MHC I loci (*Aime*-C, *Aime*-F, *Aime*-I, and *Aime*-L) and six MHC II loci; (**b**) SuHaII, including six MHC II loci (DQA1, DQA2, DQB1, DQB2, DRB3, and DRA); (**c**) DQ, including DQB1, DQB2, DQA1, and DQA2; and, (**d**) DR, including DRB3 and DRA; (**e**–**h**) Four individual loci.

The model-based analysis confirmed the randimization results. We found a negative relationship between allele sharing females and males and natural mating success at SuHa, SuhaII, DQ, and DR. Separately examining the MHC loci revealed that the overall negative trend might have been caused by lower allele sharing at DQA1, DQA2, DQB1, and DRB3 between the mated pairs (Figure 6). A positive relationship was found between the functional amino acid distance of females to males and natural mating success at SuHa, SuhaII, and DQ, and at three individual loci (DQA1, DQA2, and DQB1; Table 4). The model-based analysis indicated that higher natural mating success resulted from more MHC-dissimilar mating pairs. These results support the MHC genetic compatibility hypothesis.

### 3.3. Sperm-Egg Recognition

We successfully assigned 16 fathers to 80 offspring, including 30 twins. In 65 cases, adult females accepted sperm from 1–4 adult males, including males that are involved in natural mating and artificial insemination. 

#### 3.3.1. Observed Zygotes (Offspring) Versus Randomly Assigned Zygotes

**Figure 7:**
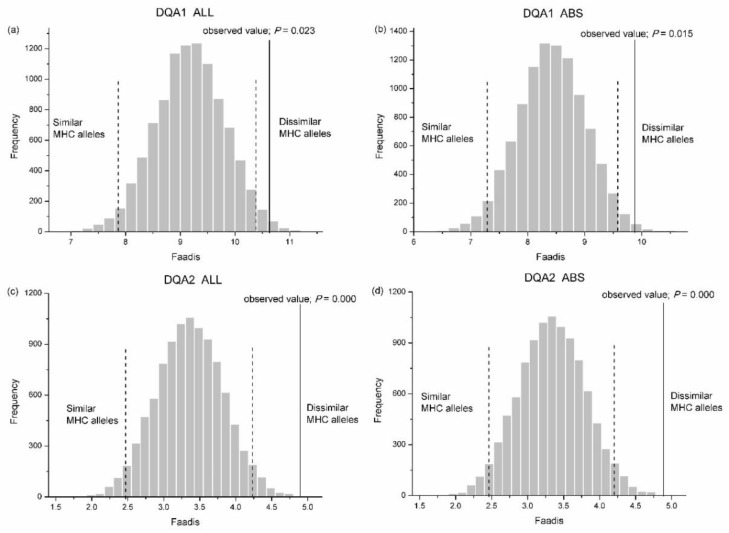
Frequency distributions of functional amino acid distances (Faadis) between eggs and randomly assigned sperm after 10,000 simulations. (**a**) whole exon2 of *Aime*-DQA1; (**b**) antigen binding site (ABS) within exon2 of *Aime*-DQA1; (**c**) whole exon2 of *Aime*-DQA2; and, (**d**) ABS within exon2 of *Aime*-DQA2. Two-tailed 95% confidence intervals are indicated by black dashed lines. MHC, major histocompatibility complex.

We found no significant difference between the observed zygotes and the randomly assigned zygotes in Faadis (egg-to-sperm MHC divergence) at all types of super haplotype and all MHC loci, except for DQA1 and DQA2 (Table S2). The Faadis of the observed zygotes at DQA1 and DQA2 was outside the 97.5% hypothesis distribution with respect to all sites and the ABS sites (Figure 7, Table S2). 

#### 3.3.2. Observed Zygotes (Offspring) Versus Other Zygotes

**Figure 8:**
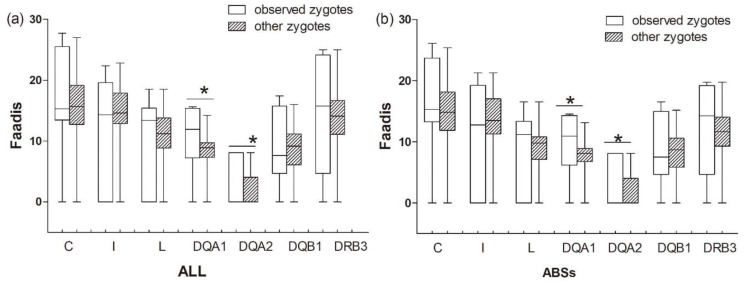
Functional amino acid distance (Faadis) for genetic compatibility at seven major histocompatibility complex loci between observed zygotes and other zygotes. (**a**) functional amino acid distance of all sites; and, (**b**) functional amino acid distance of antigen binding sites (ABSs). Asterisk shows results with *p* values that are smaller than 0.05.

The results between the observed zygotes and other zygotes (combinations of egg-to-sperm haplotypes, except for offspring haplotypes)) were similar to those between the observed zygotes and randomly assigned zygotes (Table 5, Figure 8). Observed zygotes had higher Faadis values at DQA1 and DQA2 than other zygotes at all sites and ABS sites (*P*_DQA1-ABS_ = 0.008, *P*_DQA1-ALL_ = 0.012, *P*_DQA2-ABS_ = 0.000, and *P*_DQA2-ALL_ = 0.000; Figure 8).

#### 3.3.3. Relationship between Breeding Success and MHC Divergence of Zygotes

The model-based analysis revealed that a large MHC functional amino acid distance between eggs and sperm at DQ (mainly at DQA1 and DQA2) and *Aime*-C resulted in higher breeding success, indicating the preference for maximum MHC divergence between eggs and sperm (Table 6). 

### 3.4. Mother-Fetus Recognition

**Figure 9:**
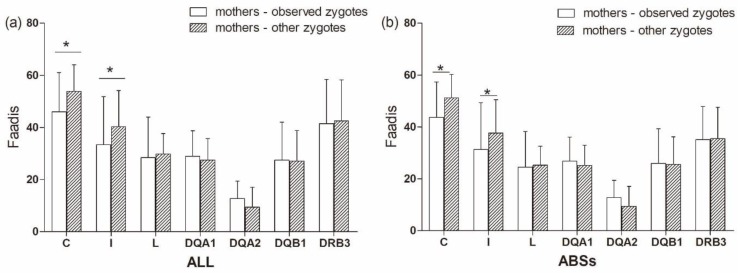
Functional amino acid distances (Faadis) at seven major histocompatibility complex loci between mothers to observed zygotes (offspring) and between mothers to other zygotes. (**a**) functional amino acid distances at all sites; and, (**b**) functional amino acid distances at antigen binding sites (ABSs). Asterisk shows results with *p* values that are smaller than 0.05.

There was no significant difference in Faadis between the observed zygotes and their mothers and other zygotes and their mothers at all super haplotypes and all loci, except for *Aime*-C and *Aime*-I (Figure 9, Table S3). The observed zygotes had significantly lower Faadis to mothers at *Aime*-C and *Aime*-I than other zygotes (*p* < 0.05, Figure 9). 

We did not find a significant relationship between breeding success and the functional amino acid distance of mothers to a combination of egg and sperm haplotypes at any MHC loci (Table S4). 

## 4. Discussion

### 4.1. Female Choice at the Inter-Individual Recognition Level

We found that female giant pandas preferred males with MHC-heterozygous and MHC-dissimilar genotypes, which supports the heterozygosity advantage and disassortative choice of the compatibility hypotheses, respectively. It has been proposed that females usually use male ornaments as a cue for choosing MHC-heterozygous males, and olfaction as a cue for choosing MHC-dissimilar males [15]. Body color varies among giant pandas, and it may reflect condition [73,74,75]. Therefore, we propose that female giant pandas assess males’ physical condition in order to choose MHC-heterozygous mates by evaluating the depth of body color. During the mating season, giant pandas secrete musk from their anal glands and in their urine, which is highly odorous [43], thus it is possible that females use odor to choose among male MHC genotypes. Animals can simultaneously use two cues, and the two strategies are not mutually exclusive, particularly in species with both good vision and olfaction [19,76,77]. A recent study on giant pandas reported multimodal signal behavior between females and males, including olfaction, vision, and hearing [55], supporting the use of two strategies. It has been reported that female pandas use odor cues prior to face-to-face meetings, so we propose that females choose MHC-dissimilar mating partners based on odor cues alone, and they use body color depth to visually choose MHC-heterozygous males. A “multiple-cue strategy” was also found in a study of female mice that changed strategy when the males’ urinary scent-marking rate changed [78], and in lizards that use coloration as a mate choice cue at long distances, but use odor at short distances [79]. Using multiple cues to choose a mate may be more common than expected, as females gain more information and reduce their selection costs [80]. 

The preference for MHC-dissimilar mates might be due to inbreeding avoidance in giant panda populations. In general, if mate choice aims to avoid inbreeding, then we expected to see significantly lower relatedness in the observed pairs than in randomly assigned pairs [20,63,81]. However, our results revealed no significant difference between observed pairs and randomly assigned pairs (Table 1), suggesting that female giant pandas do not attempt to avoid inbreeding. Avoiding inbreeding may not be as important to females as maximizing the number of MHC polymorphisms in their offspring in order to resist pathogens. In addition, we did not find any evidence for the heterozygote advantage hypothesis that is based on the MLH and *d*^2^ results, suggesting that overall genetic diversity has no effect on mate choice. These findings indicate that female mate choice targets functional MHC genes rather than other regions, or it is a byproduct of inbreeding avoidance. 

Some studies have reported that giant pandas are susceptible to parasites and viruses [82,83,84,85], suggesting that the MHC genes are important in female mate choice at the inter-individual recognition level. Furthermore, our results show that females favored partners that were the most MHC-dissimilar to themselves, resulting in offspring with high heterozygosity. Whether offspring have high immunocompetence should be addressed in future studies regarding the relationship between MHC heterozygosity and immunocompetence in the giant panda. 

### 4.2. Cryptic Female Choice at the Sperm-Egg Recognition Level

Female giant pandas usually mate with multiple males, possibly to increase fertilization success and offspring genetic quality, as has been found in other species [3,86,87]. Our results revealed that sperm and eggs do not randomly combine, and that sperm from zygotes observed was more dissimilar to eggs at DQA1 and DQA2 than sperm from other zygotes (Figure 7 and Figure 8 and Table 5). Two mechanisms may explain this: cryptic female choice or sperm competition [15,16,20], but we could not identify which was the most important. Nevertheless, combined sperm and eggs had the maximum functional amino acid distance, which supports the female MHC-disassortative choice of compatibility hypothesis that is described above. 

### 4.3. Mother-Fetus Recognition Level

The observed zygotes were more similar to mothers at *Aime*-C and *Aime*-I than the randomly assigned zygotes and other zygotes (Figure 9). These findings suggest that the observed zygotes had higher compatibility at *Aime*-C and *Aime*-I, which could decrease or block graft rejections from the mother’s immune system. MHC class I molecules play an important role in the immune reaction between the mother and fetus, e.g., HLA-G molecules are present at the mother-child interface of trophoblastic cells and protect the fetus from the lytic activity of maternal uterine natural killer cells [88,89]. The identification of an HLA-G ortholog in the giant panda would elucidate the effects of mother-fetus immunity in this species; however, MHC class I genes have similar loci, making HLA-G orthologs difficult to identify in the giant panda. 

### 4.4. Hierarchical and Cooperative Effects

From the super haplotype level to individual loci, female mate choice was hierarchical in nature. For example, we found that SuHa, which represents all of the functional MHC genes in giant pandas, predicted MHC-heterozygous female choice. However, when separately analyzing SuHaI and SuHaII, the effect could only be observed in SuHaII. When we then excluded DR from SuHaII and analyzed DQ alone, the heterozygosity advantage could still be detected. In contrast, the heterozygosity advantage could not be detected when only analyzing DR. Finally, when we focused on the individual locus level, only one out of three DQ genes (DQB1) still predicted female choice. A similar pattern was found for the genetic compatibility hypothesis. 

Alternatively, multiple MHC genes that act cooperatively can explain the above example. The effect of DQ seemed to be larger than that of DQB1 alongside DQA1 and DQA2. The integration of the loci or super haplotypes had a greater effect than the sum of the loci or super haplotypes. 

The SuHa results were inconsistent with those of SuHaII with respect to inter-individual recognition, suggesting that SuHaII is more important than SuHaI. This may have been caused by greater variation in SuHaII than in SuHaI. 

### 4.5. Relative Importance of MHC-I, MHC-II, DQ, and DR

MHC class I, MHC class II, DQ, and DR were differentially important at the inter-individual, gamete, and mother-fetus recognition levels. More MHC II genes predicted compatible female mate choice than the MHC I genes, e.g., naturally mated males were more dissimilar to their partners at SuHaII but not at SuHaI. Furthermore, all four of the MHC class II genes (DQA1, DQA2, DQB1, and DRB3) predicted compatible female mate choice, while only one out of three MHC class I genes had a significant result. At the gamete level, the sperm were more dissimilar to eggs at DQ than at DR. At the mother-fetus recognition level, the observed zygotes (offspring) were more similar to their mothers at MHC class I genes, but not at MHC class II genes. 

Genes that predict female mate choice or gamete selection may be more important than other MHC loci that are involved in pathogen resistance, as revealed by many studies in which distinct molecules that are coded by different alleles recognize specific pathogens, and their ability to resist pathogens differs [11,12,13,14], such as the DQ region. The DQ region in the giant panda differs to that in other mammals, because it contains more genes and alleles than the DR region [59]. DRB3 exhibited the most variation among the seven individual polymorphic loci. MHC polymorphisms may be driven by sexual selection [10], which is in line with our results at DQ and DRB3.

Our findings suggest that MHC loci do not play equal roles in female mate choice at the inter-individual recognition level or at other levels, and that targeted MHC loci may be key for female mate choice at the individual recognition level. Therefore, more MHC loci should be isolated and large MHC regions surveyed. 

## 5. Conclusions

Our results for *Aime*-C, *Aime*-I, and DQ support the heterozygosity hypothesis, while the results for *Aime*-C, DQ, and DR support the genetic compatibility hypothesis. Comparisons of combined sperm and other sperm-to-eggs revealed that sperm competition or *Aime*-DQA1- and DQA2-associated gamete selection occurred. The comparison of zygotes observed (offspring) and other zygotes revealed the possible *Aime*-C- and *Aime*-I-associated maternal immune tolerance mechanisms. We suggest that captive breeding programs should consider the MHC constitution. Our study provides a good foundation for studying the relationships between the MHC constitution, individual fitness (lifetime reproductive success), and mate choice cues in the giant panda.

## Figures and Tables

**Table 1:** Microsatellite results from randomization and paired Student *t*-tests.

		Compatibility Test	Heterozygote Advantage Test	
		Relatedness	MLH	*d* ^2^
Randomization	Observed	−0.087	0.751	25.961
Test ^a^	Simulated mean	−0.065	0.744	27.491
	95% CI	[−0.090, −0.040]	[0.713, 0.775]	[24.319, 30.648]
	*P*	0.079	0.651	0.345
Paired test ^b^	Potential fathers	−0.093	0.782	25.157
	Other males	−0.061	0.741	27.480
	Statistics	*t* = 1.592	Z = 0.556	*t* = 0.913
	*P*	0.115	0.578	0.364

^a^ Comparison between the natural mating group and the randomly assigned group. ^b^ Comparison between the natural mating group and the non-mating group.

**Table 2:** Functional amino acid distances for female genetic compatibility between the observed pairs and randomly assigned pairs.

Locus	Region	Simulated Mean [95% CI]	Observe Mean	*P*
SuHa	ABS	123.873 [121.759, 125.899]	127.832	**0.001**
	ALL	136.412 [134.088, 138.677]	140.728	**0.000**
SuHaI	ABS	93.169 [90.939, 95.359]	94.326	0.297
	ALL	101.310 [98.883, 103.635]	102.285	0.416
SuHaII	ABS	73.374 [71.216, 75.579]	78.033	**0.000**
	ALL	82.600 [80.143, 85.028]	87.462	**0.000**
DQ	ABS	53.815 [51.715, 55.876]	58.000	**0.000**
	ALL	57.319 [55.059, 59.577]	61.433	**0.001**
DR	ABS	45.051 [43.434, 46.662]	47.498	**0.002**
	ALL	53.531 [51.588, 55.516]	56.510	**0.003**
C	ABS	60.340 [58.005, 62.674]	62.840	**0.036**
	ALL	63.839 [61.359, 66.265]	66.431	**0.038**
I	ABS	46.787 [44.406, 49.218]	45.218	0.196
	ALL	50.388 [47.956, 52.819]	48.681	0.172
L	ABS	33.315 [31.085, 35.588]	32.624	0.548
	ALL	39.862 [37.267, 42.449]	38.584	0.340
DQA1	ABS	32.307 [30.740, 33.938]	35.587	**0.000**
	ALL	35.121 [30.740,33.938]	38.400	**0.000**
DQA2	ABS	11.596 [10.674, 12.542]	13.076	**0.001**
	ALL	11.603 [10.674, 12.542]	13.076	**0.001**
DQB1	ABS	32.821 [30.624, 35.005]	36.002	**0.005**
	ALL	34.555 [32.231, 36.817]	37.924	**0.003**
DRB3	ABS	45.051 [43.434, 46.662]	47.498	**0.002**
	ALL	53.531 [51.588, 55.516]	56.510	**0.003**

Note: Bolded figures indicate a significant difference after false discovery rate correction. ABS, antigen binding site.

**Table 3:** Mean functional amino acid distances at five super haplotypes in naturally mated pairs and non-mating pairs.

Locus		Natural Mated Pairs	Non-Mating Pairs	Statistics	*P*
SuHa	ABS	126.209	119.677	t = 3.310	**0.001**
	ALL	138.990	132.018	t = 3.298	**0.001**
SuHaI	ABS	92.315	90.284	t = 1.054	0.294
	ALL	100.215	98.262	t = 0.932	0.353
SuHaII	ABS	77.548	70.363	t = 3.960	**0.000**
	ALL	86.828	79.532	t = 3.630	**0.000**
DQ	ABS	57.961	50.322	Z = −4.604	**0.000**
	ALL	61.375	53.769	Z = −4.435	**0.000**
DR	ABS	46.879	44.284	t = 2.298	**0.024**
	ALL	55.732	52.649	t = 2.235	**0.027**

Note: Bolded figures indicate a significant difference after false discovery rate correction. ABS, antigen binding site.

**Table 4:** Association between natural mating success and major histocompatibility complex functional amino acid distances of females and males.

	ABS		ALL	
	F	*P*	F	*P*
SuHa	9.004	**0.003**	8.518	**0.004**
SuHaI	1.241	0.266	0.919	0.338
SuHaII	10.933	**0.001**	8.634	**0.003**
DQ	11.649	**0.001**	10.11	**0.002**
DR	3.33	0.068	2.861	0.091
C	3.849	0.050	3.498	0.062
I	0.406	0.524	0.475	0.491
L	0.122	0.727	0.469	0.494
DQA1	10.915	**0.001**	9,465	**0.002**
DQA2	4.688	**0.031**	4.688	**0.031**
DQB1	6.953	**0.009**	6.88	**0.009**
DRB3	3.33	0.068	2.861	0.091

Note: There were 954 degrees of freedom for each genetic variable tested. Bolded figures indicate a significant difference after false discovery rate correction. ABS, antigen binding site.

**Table 5:** Mean functional amino acid distances at five super haplotypes in observed zygotes and other zygotes.

Locus		Observed Zygotes	Other Zygotes	Statistics	*P*
SuHa	ABS	31.119	31.153	Z = −1.184	0.237
	ALL	34.056	34.383	t = −0.228	0.820
SuHaI	ABS	22.290	23.649	Z = −0.096	0.924
	ALL	24.082	25.684	Z = −0.027	0.978
SuHaII	ABS	19.959	18.785	Z = −1.696	0.090
	ALL	22.136	21.261	Z = −1.108	0.268
DQ	ABS	15.164	13.717	Z = −2.624	**0.009**
	ALL	15.997	14.677	Z = −2.526	**0.012**
DR	ABS	11.857	11.633	Z = −0.304	0.761
	ALL	13.924	13.943	Z = −0.023	0.982

Note: Bolded figures indicate a significant difference after false discovery rate correction. ABS, antigen binding site.

**Table 6:** Association between breeding success and major histocompatibility complex functional amino acid distances between egg and sperm haplotypes.

		ABS		ALL	
	*df*	F	*P*	F	*P*
SuHa	472	0.671	0.413	0.989	0.320
SuHaI	467	1.827	0.177	2.072	0.151
SuHaII	453	0.531	0.466	0.120	0.730
DQ	453	0.001	0.982	12.32	**0.000**
DR	344	0.007	0.935	0.101	0.751
C	283	0.205	0.651	0.135	0.714
I	256	5.440	0.020	5.749	0.017
L	252	0.902	0.343	1.136	0.288
DQA1	**399**	7.058	**0.008**	6.325	**0.012**
DQA2	**146**	16.090	**0.000**	16.09	**0.000**
DQB1	242	0.107	0.743	0.072	0.788
DRB3	344	0.007	0.935	0.101	0.751

Note: Bolded figures indicate a significant difference after false discovery rate correction. ABS, antigen binding site.

## Supplementary Materials

The following are available online at https://www.mdpi.com/2073-4409/8/3/257/s1. Figure S1, Allele linkage relationships of six major histocompatibility complex (MHC) class II genes for SuHaII. Asterisk indicates recombinational SuHaII. Figure S2, Allele linkage relationships of four major histocompatibility complex (MHC) class I genes for SuHaI. Figure S3, Allele linkage relationships between SuHaI and SuHaII for SuHa. Figure S4, Allele linkage relationships of DQ genes. Asterisk indicates recombinational DQ super haplotype. Table S1, Genetic variation in major histocompatibility complex (MHC) class I and class II molecules and super haplotypes. Table S2, Functional amino acids in observed zygotes and randomly assigned zygotes. Table S3, Mean functional amino acid distances at five super haplotypes between mothers and observed zygotes and between mothers and other zygotes. Table S4, Association between breeding success and major histocompatibility complex (MHC) functional amino acid distances of the mother and a combination of egg and sperm haplotypes.
