# Supplementary material for: Reproductive Strategy Inferred from Major Histocompatibility Complex-Based Inter-Individual, Sperm-Egg, and Mother-Fetus Recognitions in Giant Pandas (Ailuropoda melanoleuca)
Source: Cells. 2019 Mar 19;8(3):257. doi: 10.3390/cells8030257 (PMC6468540; doi:10.3390/cells8030257)
Supplement: Supplementary file 1 [file cells-08-00257-s001.zip › Table S1-S4.docx]

|  | Variable AA% |  | Mean dif AA |  |
| --- | --- | --- | --- | --- |
|  | ABS | ALL | ABS | ALL |
| C | 29.8 | 12.6 | 7.679 | 9.214 |
| I | 28.1 | 12.1 | 7.952 | 10.047 |
| L | 19.3 | 9.9 | 4.133 | 6.867 |
| DQA1 | 31.3 | 9.7 | 2.952 | 4.476 |
| DQA2 | 6.3 | 1.2 | 0.667 | 0.667 |
| DQB1 | 36.8 | 10.1 | 3.400 | 4.600 |
| DRB3 | 89.5 | 30.3 | 7.429 | 11.810 |
| SuHa | 31.5 | 11.9 | 28.816 | 39.881 |
| SuHaI | 29.8 | 11.7 | 21.300 | 27.839 |
| SuHaII | 44.3 | 13.2 | 11.634 | 16.601 |
| DQ | 25.5 | 7.1 | 5.575 | 7.837 |
| DR | 89.5 | 30.3 | 7.429 | 11.810 |

**Table** **S1** Genetic variation in major histocompatibility complex (MHC) class I and class II molecules and super haplotypes.

Note: “Variable AA%” shows the percentage of variable amino acid and “Mean diffAA” denote mean value of different number of amino acids between pairwise alleles or superhaplotypes.

**Table S2** Functional amino acids between observed zygotes and randomly assigned zygotes.

| Locus |  | Simulated mean | Observed | *P* |
| --- | --- | --- | --- | --- |
|  |  | [95%CI] | mean |  |
| SuHa | ABS | 31.151 [29.248, [32.832] | 31.119 | 0.970 |
|  | ALL | 34.280 [32.170, 36.148] | 34.056 | 0.823 |
| SuHaI | ABS | 23.574 [21.684, 25.302] | 22.235 | 0.143 |
|  | ALL | 25.564 [23.522, 27.418] | 24.028 | 0.119 |
| SuHaII | ABS | 19.103 [17.334, 20.772] | 19.959 | 0.336 |
|  | ALL | 21.495 [19.463, 23.420] | 22.136 | 0.931 |
| DQ | ABS | 14.171 [12.626, 15.606] | 15.164 | 0.182 |
|  | ALL | 15.119 [13.466, 16.645] | 15.997 | 0.262 |
| DR | ABS | 11.688 [10.099, 13.241] | 9.858 | 0.831 |
|  | ALL | 13.909 [11.919, 15.854] | 10.627 | 0.989 |
| C | ABS | 15.551 [13.681, 17.381] | 15.418 | 0.886 |
|  | ALL | 16.475 [14.452, 18.448] | 16.246 | 0.822 |
| I | ABS | 11.781 [9.949, 13.558] | 10.395 | 0.129 |
|  | ALL | 12.760 [10.868, 14.590] | 11.256 | 0.111 |
| L | ABS | 8.809 [7.688, 9.902] | 8.397 | 0.469 |
|  | ALL | 10.427 [9.111, 11.728] | 9.799 | 0.349 |
| DQA1 | ABS | 8.421 [7.256, 9.557] | 9.858 | **0.015** |
|  | ALL | 9.179 [7.923, 10.401] | 10.627 | **0.023** |
| DQA2 | ABS | 3.360 [2.491, 4.234] | 4.857 | **0.000** |
|  | ALL | 3.360 [2.491, 4.234] | 4.857 | **0.000** |
| DQB1 | ABS | 8.390 [6.987, 9.805] | 8.594 | 0.777 |
|  | ALL | 8.891 [7.381, 10.404] | 9.004 | 0.879 |
| DRB3 | ABS | 11.688 [10.099, 13.241] | 11.857 | 0.831 |
|  | ALL | 13.909 [11.919, 15.854] | 13.924 | 0.989 |

Note: Bold figures show a significant difference after FDR correction.

**Table S3** Mean functional amino acid distances at five super haplotypes between mothers and observed zygotes and between mothers and other zygotes.

| Locus |  | Observed | other | statistics | *P* |
| --- | --- | --- | --- | --- | --- |
|  |  | zygotes | zygotes |  |  |
| SuHa | ABS | 90.952 | 90.097 | t=0.560 | 0.577 |
|  | ALL | 99.743 | 99.554 | t=0.111 | 0.912 |
| SuHaI | ABS | 65.409 | 65.962 | t=-0.322 | 0.749 |
|  | ALL | 70.365 | 71.457 | t=-0.596 | 0.553 |
| SuHaII | ABS | 57.081 | 56.713 | Z=-.427 | 0.669 |
|  | ALL | 63.872 | 64.214 | t=-0.174 | 0.863 |
| DQ | ABS | 41.991 | 41.322 | Z=-0.648 | 0.517 |
|  | ALL | 44.445 | 44.076 | Z=-0.434 | 0.664 |
| DR | ABS | 35.057 | 35.459 | t=-0.305 | 0.761 |
|  | ALL | 41.509 | 42.582 | t=-0.653 | 0.516 |

**Table S4** Association between breeding success and major histocompatibility complex (MHC) functional amino acid distances of the mother and a combination of egg and sperm haplotypes.

|  |  | ABS | | ALL | |
| --- | --- | --- | --- | --- | --- |
|  | *df* | F | *P* | F | *P* |
| SuHa | 472 | 2.775 | 0.096 | 3.591 | 0.059 |
| SuHaI | 467 | 3.253 | 0.072 | 3.888 | 0.049 |
| SuHaII | 453 | 1.053 | 0.305 | 1.822 | 0.178 |
| DQ | 453 | 3.453 | 0.064 | 4.742 | 0.051 |
| DR | 344 | 1.705 | 0.192 | 2.188 | 0.131 |
| C | 283 | 0.231 | 0.631 | 0.375 | 0.541 |
| I | 256 | 3.073 | 0.081 | 2.827 | 0.094 |
| L | 252 | 3.558 | 0.060 | 3.782 | 0.053 |
| DQA1 | 399 | 0.531 | 0.467 | 0.272 | 0.602 |
| DQA2 | 146 | 1.962 | 0.163 | 1.962 | 0.163 |
| DQB1 | 242 | 1.78 | 0.183 | 1.786 | 0.183 |
| DRB3 | 344 | 1.705 | 0.192 | 2.188 | 0.131 |
