# Supplementary figures and images for: Reproductive Strategy Inferred from Major Histocompatibility Complex-Based Inter-Individual, Sperm-Egg, and Mother-Fetus Recognitions in Giant Pandas (Ailuropoda melanoleuca)
Source: Cells. 2019 Mar 19;8(3):257. doi: 10.3390/cells8030257 (PMC6468540; doi:10.3390/cells8030257)

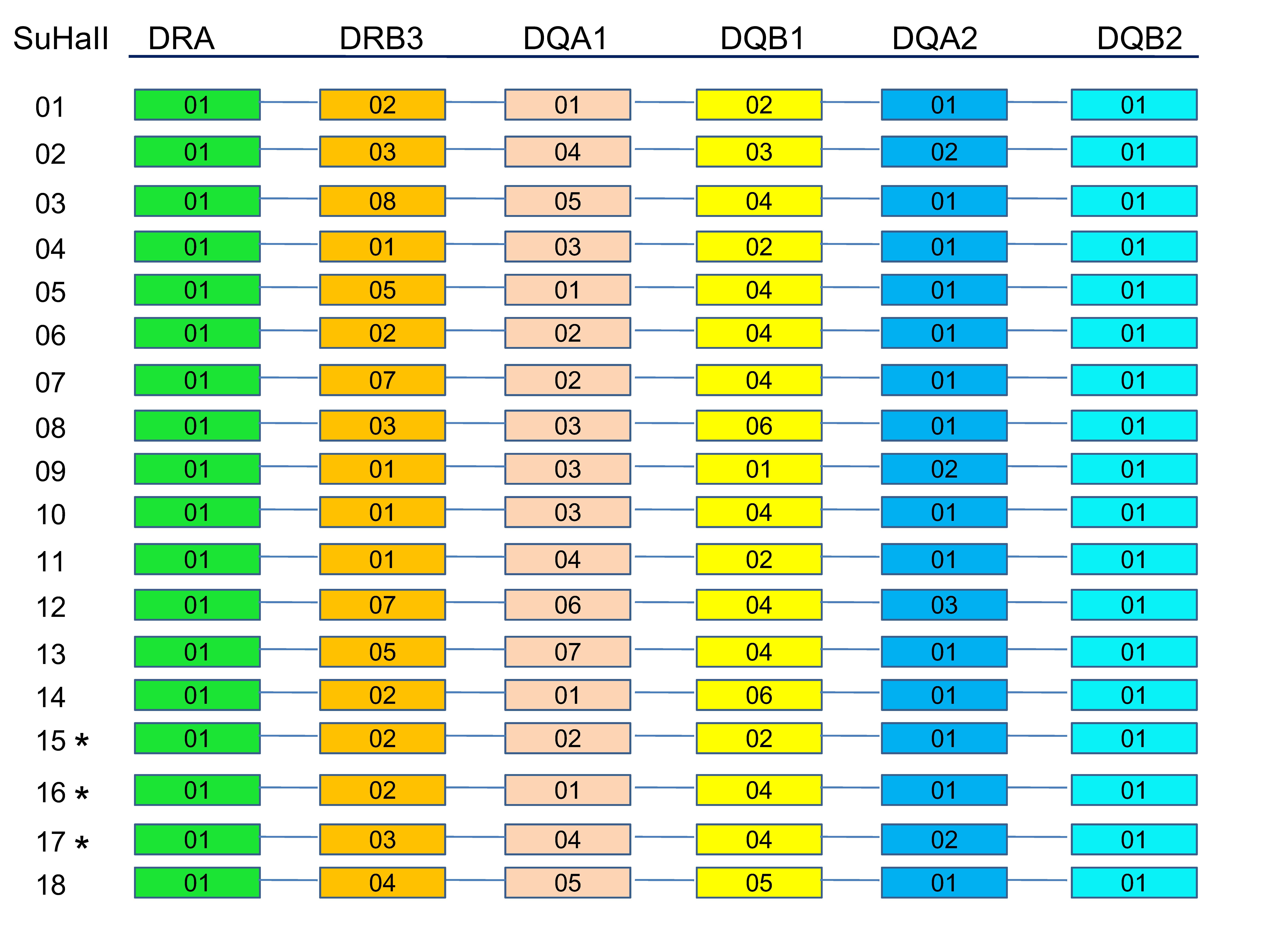

Supplement: Supplementary file 1 [file cells-08-00257-s001.zip › Fig S1.jpg]

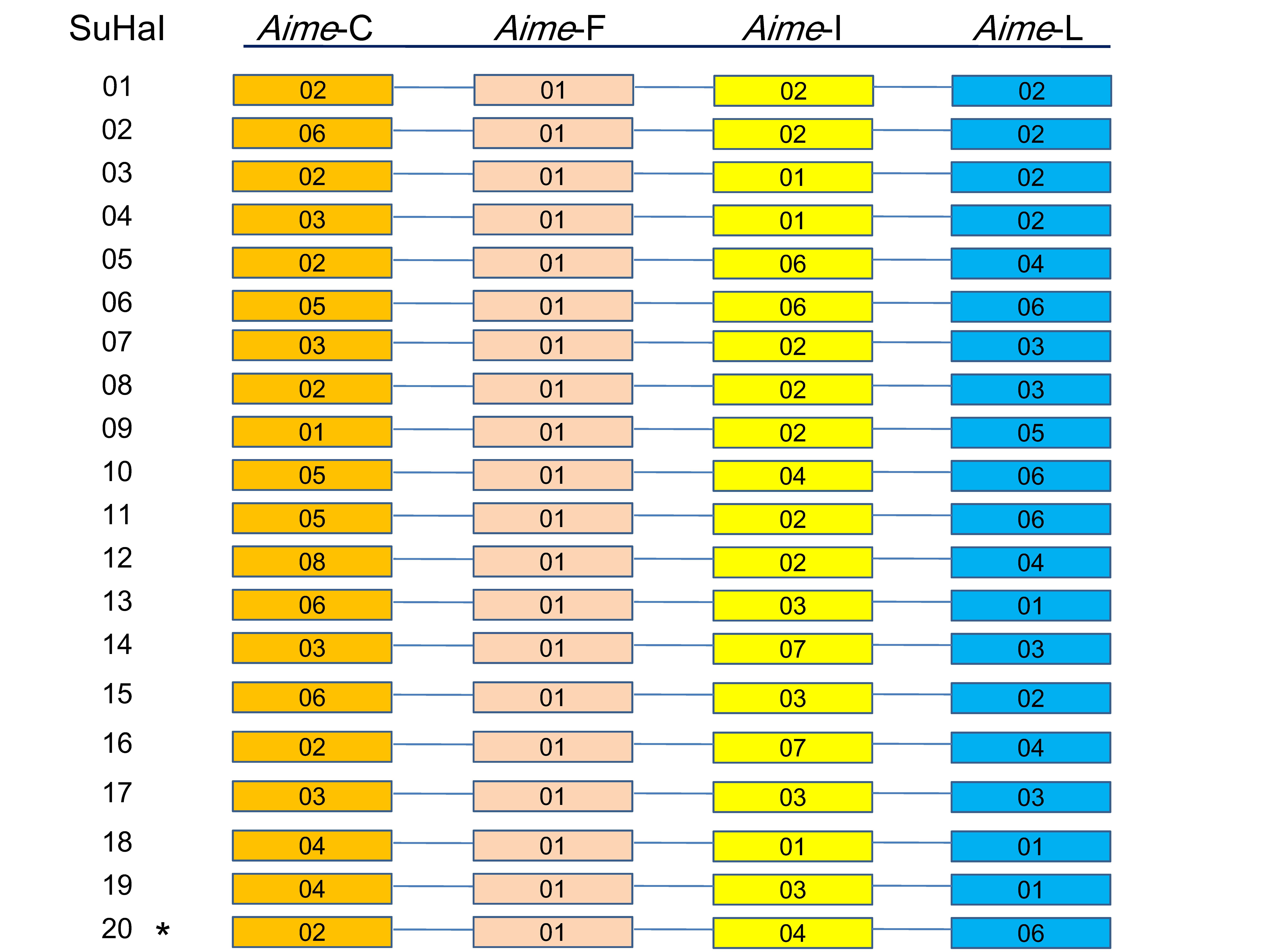

Supplement: Supplementary file 1 [file cells-08-00257-s001.zip › Fig S2.jpg]

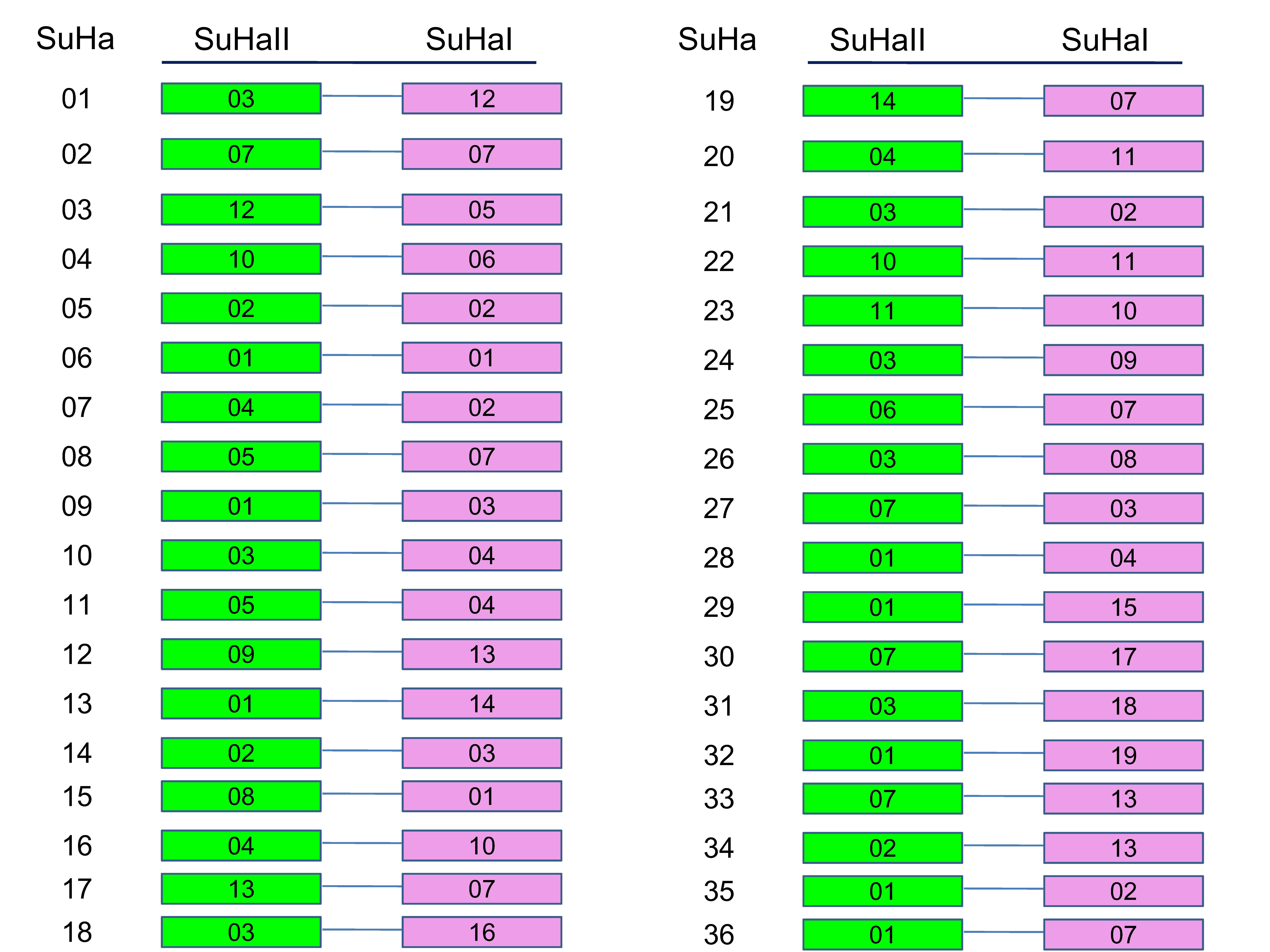

Supplement: Supplementary file 1 [file cells-08-00257-s001.zip › Fig S3.jpg]

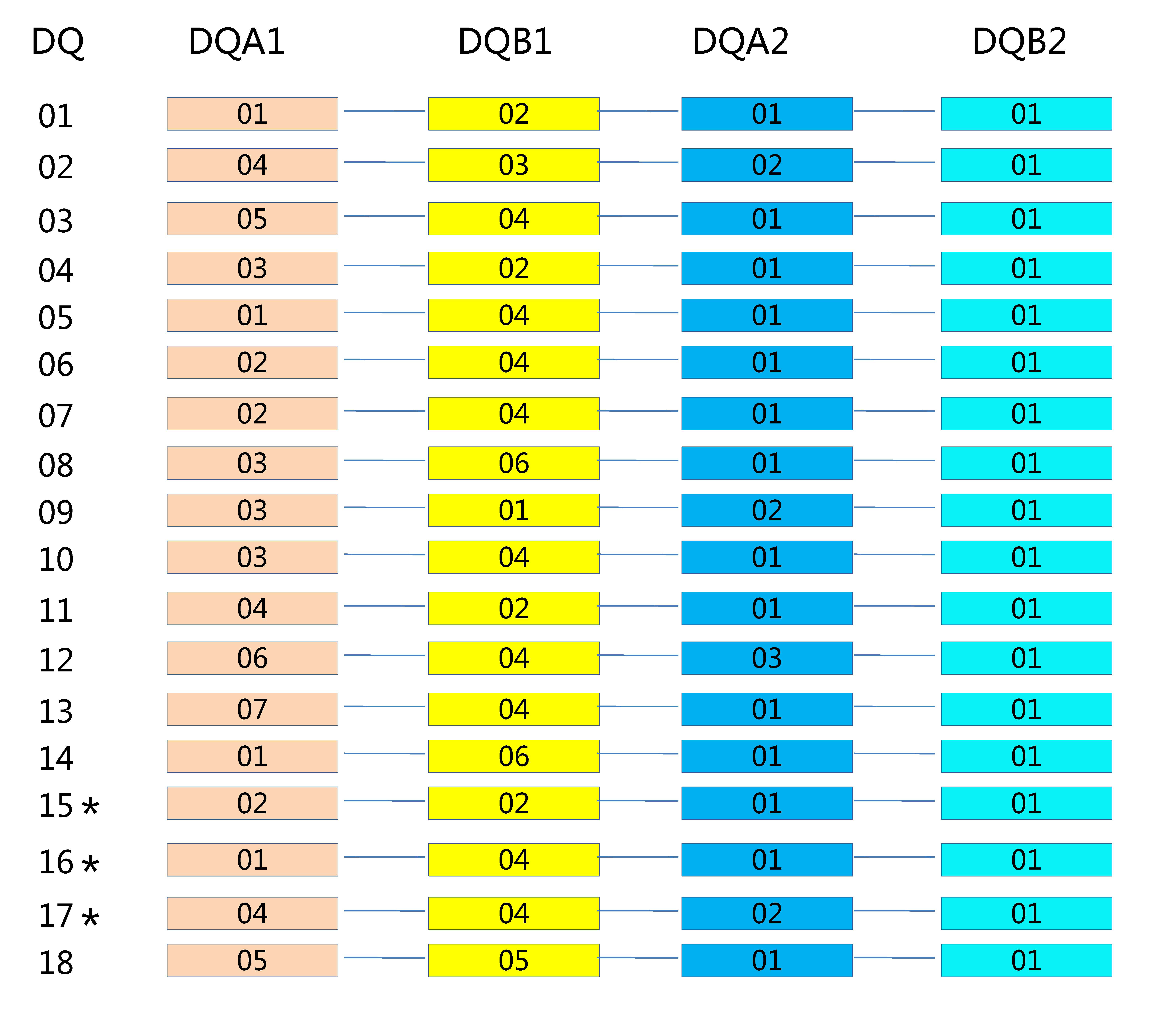

Supplement: Supplementary file 1 [file cells-08-00257-s001.zip › Fig S4.jpg]
